# An analysis of trends and distribution of the burden of road traffic injuries in Uganda, 2011 to 2015: a retrospective study

**DOI:** 10.11604/pamj.2018.31.1.15223

**Published:** 2018-09-02

**Authors:** Frederick Oporia, Angela Nakanwagi Kisakye, Rebecca Nuwematsiko, Abdulgafoor Mahmood Bachani, John Bosco Isunju, Abdullah Ali Halage, Zziwa Swaibu, Lynn Muhimbuura Atuyambe, Olive Kobusingye

**Affiliations:** 1 Department of Disease Control and Environmental Health, Makerere University School of Public Health, Kampala, Uganda; 2 Department of Health Policy Planning and Management, Makerere University School of Public Health, Kampala Uganda; 3 African Field Epidemiology Network, Lugogo House Plot 42, Lugogo Bypass, Kampala, Uganda; 4 Department of International Health and Johns Hopkins International Injury Research Unit, Johns Hopkins Bloomberg School of Public Health, Baltimore, MD, USA; 5 Department of Community Health and Behavioural Sciences, Makerere University School of Public Health, Kampala, Uganda

**Keywords:** Trends, road traffic injuries, health facilities, Uganda

## Abstract

**Introduction:**

Gobally, 1.3 million people die from road traffic injuries every year. Over 90% of these deaths occur in low-and-middle-income countries. In Uganda, between 2012 and 2014, about 53,147 road traffic injuries were reported by the police, out of which 8,906 people died. Temporal and regional distribution of these injuries is not known, hence hindering targeted interventions. We described the trends and distribution of health facility reported road traffic injuries in Uganda from 2011 to 2015.

**Methods:**

We obtained monthly data on road traffic injuries, from 112 districts from the Ministry of Health Uganda. We analyzed the data retrospectively to generate descriptive statistics.

**Results:**

A total of 645,805 road traffic injuries were reported from January 2011 through December 2015 and 2,807 deaths reported from 2011 through 2014. Injuries increased from 37,219 in 2011 to 222,267 in 2014 and sharply dropped in December 2015 to 57,149. Kampala region had the highest number of injuries and deaths (18.3% (117,950/645,805) and 22.6% (634/2807)) respectively whereas Karamoja had the lowest injuries and deaths (1.7% (10,823/645,805) and 0.8% (21/2807)) respectively. Children aged 0-4 years accounted for 21.9% (615/2807) deaths; mostly females 81% (498/615) were affected.

**Conclusion:**

Road traffic injuries increased during 2011-2014. Injuries and deaths were highest in Kampala and lowest in Karamoja region. It was noted that health facilities mostly received serious injuries. It is likely that the burden is higher but under reported. Concerted efforts are needed to increase road safety campaigns in Kampala and surrounding regions and to link pre-hospital deaths so as to understand the burden of road traffic crashes and recommend appropriate interventions.

## Introduction

Road transport provides a wide range of benefits to individuals and countries by enabling easy access to markets, goods and services. However, the safety of people on the world's roads is a major public health challenge [1]. Globally, injuries are a growing public health concern. An escalating toll of road traffic injuries (RTIs) leaves more than 1.3 million deaths on the world's roads every year and over 50 million people disabled. Over ninety percent of these RTIs occur in low-and-middle-income countries (LMICs) despite owning just about 54% of the world's motor vehicles [2]. There is an estimated global annual rate of road traffic deaths of about 17.4 per 100,000 population, a figure which is more than double in LMICs. These RTIs are predicted to move from the current 8^th^ to 6^th^ leading cause of death by the year 2030 [2]. African and the Eastern Mediterranean regions have one of the highest rates of road traffic deaths [3], despite remaining the least motorized continents [4]. For example, Africa possesses only 2% of the world's motor vehicles but contributes about 16% of global road traffic fatalities. Nigeria, Ethiopia, Tanzania and South Africa are the highest contributors after which Uganda lies in the fifth position [2,4,5]. There have been an observable annual increase of road traffic injuries in the Middle East and North African (MENA) region [5]. Nearly half of all global road traffic deaths are among pedestrians and motorcyclists, mostly occurring on urban roads [2]. Pedestrians are the most vulnerable road users, making up 22% of the annual global road traffic deaths in LMICs [2,6,7], followed by motorcyclists, pedal cyclists and car occupants [4]. In Uganda, the incidence of RTIs is steadily increasing. It is estimated that every day, there are about 190 deaths per 10,000 vehicles in Uganda, which is among the highest incidences globally [8,9]. Between 2012 and 2014, there were 53,147 RTIs reported by the Uganda police, out of which 8,906 fatalities were recorded [9]. It is believed that these figures underestimate the burden of RTIs because not all road traffic crashes are reported to the Police due to fear of criminal charges being instituted against them. Most people only report to health facilities after sustaining a road traffic injury to receive medical attention. Health facilities in all the districts in Uganda submit monthly reports to the Ministry of Health using the Health Management Information System (HMIS). These reports also include a section on road traffic injuries. However, the temporal and regional distribution of these injuries, including persons most affected, is not known, hence hindering targeted interventions. The objective of this study was to describe the trends and distribution of health facility reported RTIs in Uganda from calendar year 2011 to 2015.

## Methods

We conducted a five-year retrospective study from January 2011 to December 2015 to describe the trends and distribution of RTIs in Uganda. We used data that was collected through the HMIS reports from all the 112 districts of Uganda (as of the year 2016) and submitted electronically to the Ministry of Health (MoH). The parameters reported in the HMIS tool include category of injuries, deaths, age, sex and district. Only two age categories were provided in the HMIS dataset that was used: 0-4 years, and five years and above. Data on RTIs was extracted from the MoH database and processed using Microsoft Excel 2013. Since the HMIS tool used to capture the data is nationally standardized, there was no need to pre-test it. It was also assumed that the data were captured by trained Health Informatics personnel countrywide, as per the ministry of health requirement. Data on both fatal and non-fatal RTIs were reported in the HMIS tool. The 112 districts of Uganda were grouped into ten regions according to the 2011 Uganda Demographic Health Survey (UDHS) sites [10]. These regions are a mix of both urban and rural districts. We obtained permission to access the data from the Ministry of Health Kampala and permission to conduct the study was obtained from Makerere University School of Public Health Higher Degrees Research and Ethics Committee (HDREC). No personal identifiers were captured in the data; therefore confidentiality was maintained throughout the data abstraction and analysis processes. We conducted analysis to generate descriptive statistics on RTIs. We defined non-fatal RTIs as patients who received health care at either outpatient or inpatient departments and were discharged. Fatal RTIs were defined according to the world health organization (WHO); as patients who sought health care at a health facility and died at the facility within 30 days from the day of injury (WHO, 2013).

## Results

### Demographic characteristics of respondents

About 645,805 non-fatal RTIs were reported between January 2011 and December 2015. From January 2011 to December 2014, health facilities reported 2,807 fatal RTIs. The dataset did not indicate RTI fatalities for the year 2015. Among the non-fatal RTIs, more than half 58.4% (377,332/645,805) of the victims were males. Half of the injured victims 51.6% (32,515/63,045) were females and children under-five years accounted for 9.8% (63,045/645,805) of the victims. Among the fatal RTIs, males above 5 years were the most affected, 60.2% (1690/2807). Table 1 about here

**Table 1:** Demographic characteristics of health facility reported road traffic injuries in Uganda

Non-fatal RTIs, N= 645,805		
Variable		Frequency, (%)
Sex	Female	268,473 (41.6)
	Male	377,332 (58.4)
Age group	0-4 years	63,045 (9.8)
	5 years and above	582,760 (90.2)
	0-4 years, Male	30,530 (48.4)
	0-4 years, Female	32,515 (51.6)
	5 years and above, Male	346,802 (59.5)
	5 years and above, Female	235,958 (40.5)
**Fatal RTIs, N= 2807**		
Age group	Females, 0-4 years	498 (17.8)
	Males, 0-4 years	117 (4.2)
	Females, 5 years above	502 (17.8)
	Males, 5 years above	1690 (60.2)

### Trends of health facility reported RTIs in Uganda between 2011 and 2015

There was an increasing trend of RTIs over the five years in Uganda, with the peak recorded in calendar year 2014 (222,267) and the lowest in 2011 (37,219). The number of RTIs was steady between the year 2012 and 2013 and nearly doubled between the year 2013 and 2014, after which a sharp decrease (57,149) was observed in 2015. From 2011 to 2014, there was a rise in the number of deaths due to RTIs. Health facilities reported 2,807 deaths due to RTIs over the four-year period. Fatal RTIs increased nearly five times from 217 in the year 2011 to 1070 in 2014 Figure 1. In 2014, the number of RTIs was highest in Kampala region whereas there were generally fewer injuries in 2015 Figure 2.

**Figure 1:**
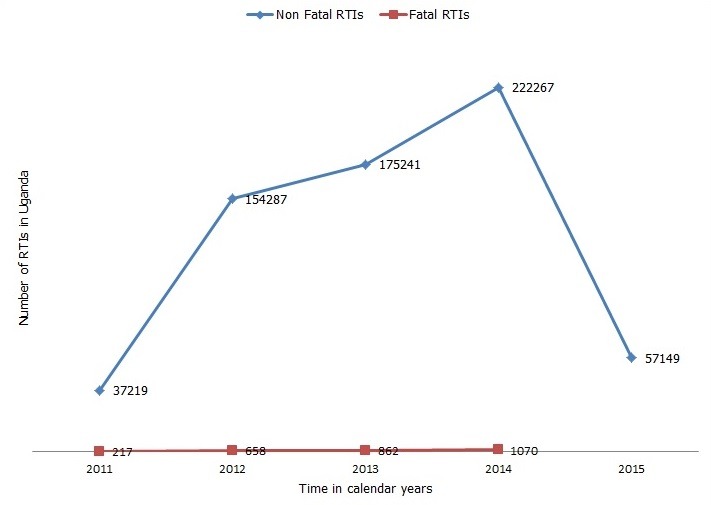
Trends of health facility reported road traffic injuries in Uganda, 2011-2015

**Figure 2:**
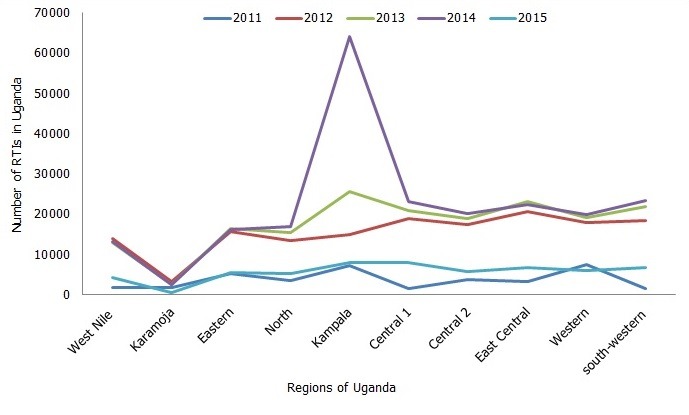
Trends of non-fatal road traffic injuries by region, 2011-2015

### Distribution of non-fatal and fatal RTIs in Uganda

**Figure 3:**
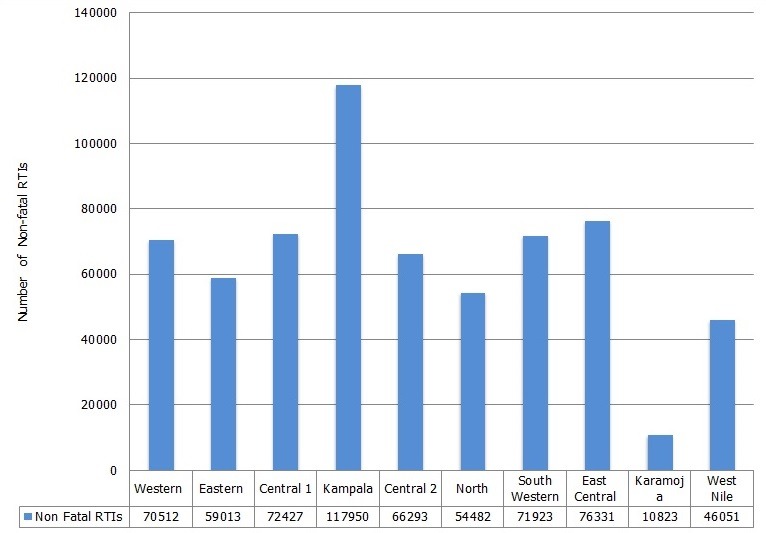
Distribution of non-fatal road traffic injuries by region, 2011-2015

**Figure 4:**
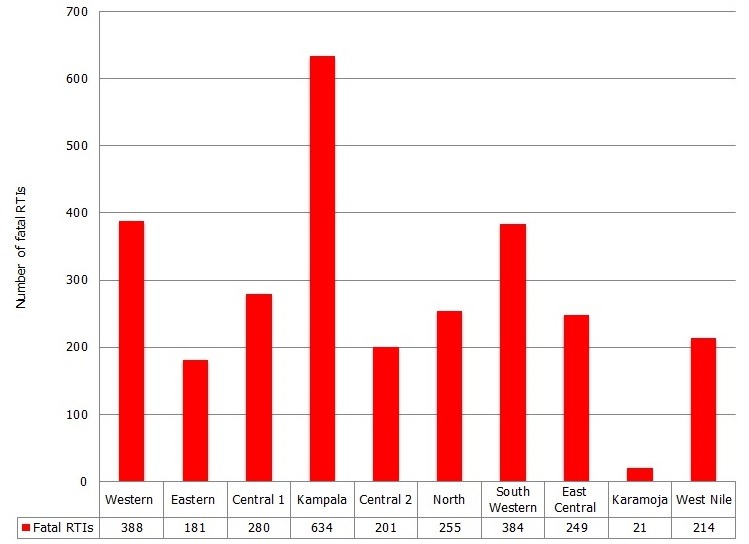
Distribution of fatal road traffic injuries by region, 2011-2014

The majority 85.39% (551,437/645,805) of the RTIs were at Outpatient Department (OPD), and 14.61% (94,368/645,805) at Inpatient Department (IPD). The highest number of RTIs were recorded in Kampala region, followed by East Central region (18.26% (117,950/645,805) and 11.8% (76331/645,805)) respectively. Karamoja region had the lowest number of RTIs 1.7% (10823/645,805) Figure 3. Among the fatal RTIs, Kampala region recorded the highest proportion of 22.59% (634/2807), followed by Western 13.82% (388/2807) and South Western regions 13.68% (384/2807) respectively. Karamoja region had the least number of road traffic deaths reported 0.75% (21/2807) Figure 4.

## Discussion

Results from this study indicate that on average, there are about 129,161 non-fatal RTIs and about 702 fatal RTIs every year in Uganda. Throughout the five years, Kampala region reported the highest number of both fatal and non-fatal RTIs while Karamoja region reported the lowest number of RTIs. We found that there were generally more RTIs among males compared to the females. A possible explanation for the high number of injuries among males is that they are more common on the roads, and therefore are more likely to be involved in RTIs. Males are also more risk takers than females, a behaviour that makes them more likely to be involved in road traffic crashes [11]. Our results are consistent with other studies that found males being most affected by road traffic injuries than females [3,12-14]. It should be noted that a sizable number of women were also involved in RTIs. This could be due to the fact that these were health facility reported injuries and females have better health seeking behaviour than males and therefore are most likely to report to a health facility. It should also be noted that the annual average number of fatal and non-fatal health facility reported RTIs is higher than what was reported by the police in the previous years of 2012 to 2014. This discrepancy could be associated with the fear of reporting RTIs to the police because many victims are afraid of possible conviction. Almost 10% of RTI victims from 2011 to 2014 were children below the age of five years. The high number of children who experienced RTIs could be because parents and caretakers of this age group take their children to health facilities whenever they get involved in any kind of injury. The high fertility rate in Uganda could also mean that the pool of children at risk of RTIs is large. Fifty six percent of the Ugandan population is below the age of 18 years, and about 19% are below five years [15] . Although most children below five years are not involved in activities that would expose them to the road, Uganda's road network is characterized by motorized traffic passing through or in close proximity to densely populated areas. The poor public transport also forces parents of children below five years to carry them on commercial motorcycles or bicycles, further exposing them to the risk of road traffic crashes. Public transport vehicles have no child restraints, and child restraint use even in private vehicles is rare. This vulnerability is not limited to Uganda. A hospital based study in Pakistan [16] found that injury was the third leading cause of death among under-fives. Studies conducted in Tanzania and Venezuela revealed that RTIs were a common cause of morbidity and mortality [14,17].

There was an increasing trend of RTIs and deaths during the five year period. The highest number of road traffic injuries that was reported in 2014 could be associated with the fact that Uganda has a rapidly growing population, that is simultaneously rapidly motorizing and urbanizing [15]. The largest category of motor vehicles joining the fleet daily is commercial motorcycles and they are associated with high rates of crashes, injuries and deaths [18]. The upward trend could therefore be a reflection of the growing population, accompanied by an expanding fleet predominated by high-risk motorcycles. It is estimated that 1,000,000 vehicles were imported into Uganda between 2009 and 2011 [15]. Between 2011 and 2015, a number of roads were constructed and tarmacked, which might have led to over speeding, hence more road traffic crashes. It could also be that people have adopted better health seeking behaviours because of several health education and road safety campaigns in Uganda. The decrease in the number of road traffic injuries in 2015 could be due to the road safety campaigns by the Uganda police such as the “Fika salama” (arrive safely) which was launched in 2015. This may have made people more cautious on roads. Similar to other studies in other countries, a decline in the number of RTIs has been recorded. For example, a seven-year trends study in Iran noted a decrease in number of RTIs [19]. Although this study shows a very high number of road traffic injuries compared to the Uganda Police Annual Crime Reports of 2011 to 2014, there is a general agreement on the fact that there is a rising trend of road traffic crashes in Uganda [9]. On the other hand, some studies in other countries have shown an increasing trend of RTIs [20-23]. Disparities in the number of RTIs were between predominantly urban regions and rural regions. For example, injuries were most frequent in Kampala and lowest in Karamoja region during the study period. A number of factors could be attributed to these disparities. The high number of injuries in Kampala could be due to the fact that Kampala is the capital city, most densely populated and the most motorized region in Uganda [15]. It could also be because health units are more accessible and people can afford to go for treatment in case of any injury. Also noteworthy is that, Kampala has some of the busiest roads and therefore the crashes that occur mostly result into a need for hospital care.

Predominantly, rural and less motorized Karamoja region had the lowest number of road traffic injuries. It was also not surprising that other less motorised regions such as East and Central 2 had fewer deaths from RTIs. This finding is consistent with the Annual Crime Report of the Uganda Police that found road traffic injuries to be higher in Kampala. The Annual Crime Report of the Uganda Police also revealed that Karamoja region was the least affected by the RTIs. In some developed countries like the United States of America, studies have found similar results that indicate increase in RTIs with rise in human population and increased motorization [24,25]. It was noted that these results are different from the Police findings where more deaths were registered in Central 1 than in Kampala [9]. Never the less, these results is similar to findings from a study that was conducted in Quebec that found more motor vehicle injuries in urban areas than in rural areas [26]. However, findings of this study differ from those of a study in Pakistan [16], where most injuries occurred in rural areas than in urban areas. Noteworthy is that in Uganda, it is against the law to be a car occupant without fastening a safety belt [27]. The Road Traffic and Safety Act of 1998 has sections on mandatory use of helmets and seat belts, prohibits drunk driving, installation of speed governors and licensing of vehicles. However, there is no law on mandatory use of child restraints in motor vehicles, even though these have been proven to reduce risks of child injury and death [28]. In Kampala, hardly any vehicle with a child on board has a child restraint and drivers seem not to pay particular attention. This leaves child car occupants at greater risk of sustaining injuries while the vehicle is moving, with unacceptably increasing number of deaths. This is quite different from other developed and middle income countries where child restraint use is taken seriously [29,30]. Our study had some limitations. The dataset had some missing variables that are vital, and therefore could not allow for advanced statistical analysis beyond univariate level. The dataset only had variables such as age, sex and districts. Therefore it was not possible to adjust for possible confounders that could have influenced the results. Age was broadly categorized into “below five” and “five plus”, making finer analysis based on age impossible. The data did not have the type of road user, neither did it distinguish inpatients and the outpatients. The HMIS dataset does not include severity of injuries, or hospital length of stay, both of which have links to patient outcome. Data reported to the MoH are only from people who make it to health facilities, so it excludes people who do not go to hospitals and those who died before reaching the health facility. However, these results can be generalized to the whole country since the data that was analyzed was reported by all the health facilities in the districts of Uganda. It is believed that most people report to health facilities in case of any kind of injury, as opposed to reporting to the Police, where people are afraid of dealing with criminal charges. We could not do comparisons of RTI fatalities by year because of missing data.

## Conclusion

There was a more than five-fold increase of road traffic injuries in 2014 compared to 2011, but a decrease was observed in 2015. Injuries and deaths were highest in Kampala and lowest in Karamoja region. The older population was most affected by the RTIs, mostly males. It should be noted that health facilities mostly receive victims with serious injuries, which may have led to under reporting of RTIs in Uganda; hence it's possible the burden is bigger. The government should intensify campaigns on road safety especially in Kampala region. There is need for concerted efforts to link the RTIs reported by the police and those who die at pre-hospital to that reported by health facilities so as to better understand the burden of injuries due to road traffic crashes.

### What is known about this topic

- About eighteen thousand road traffic crashes are reported by the Uganda Police every year;
- More than half of these RTIs were previously known to be occurring in the central region of Uganda;
- These reports also indicate the mechanisms of injury and the time they occurred.

### What this study adds

- There is an increasing trend in the number of RTIs in Uganda from 2011 to 2014 and a sharp decline in 2015;
- Kampala region had the highest number of RTIs whereas Karamoja had the lowest; implying that these injuries are most common in urban and most motorized regions of Uganda;
- There is an urgent need for more intensified road safety campaigns especially in highly motorized regions such as Kampala; and more studies to better understand more risk factors for RTIs in this region. This study also highlights the discrepancy between number of RTIs reported by the police and that reported by the health facilities, indicating the urgent need to sync the data from these two institutions.

## Competing interests

The authors declare no competing interests.
